# The *APOL1* p.N264K variant is co-inherited with the G2 kidney disease risk variant through a proximity recombination event

**DOI:** 10.1093/g3journal/jkae290

**Published:** 2024-12-10

**Authors:** Christopher A Simeone, Michelle T McNulty, Yask Gupta, Giulio Genovese, Matthew G Sampson, Simone Sanna-Cherchi, David J Friedman, Martin R Pollak

**Affiliations:** Harvard Medical School, Boston, MA 02215, USA; Division of Nephrology, Department of Medicine, Beth Israel Deaconess Medical Center, Boston, MA 02215, USA; Division of Pediatric Nephrology, Boston Children's Hospital, Boston, MA 02215, USA; Kidney Disease Initiative and Medical and Population Genetics Program, Broad Institute, Cambridge, MA 02142, USA; Division of Nephrology, Department of Medicine, Columbia University Irving Medical Center, Columbia University, New York City, NY 10032, USA; Harvard Medical School, Boston, MA 02215, USA; Stanley Center, Broad Institute of MIT and Harvard, Boston, MA 02215, USA; Harvard Medical School, Boston, MA 02215, USA; Division of Pediatric Nephrology, Boston Children's Hospital, Boston, MA 02215, USA; Kidney Disease Initiative and Medical and Population Genetics Program, Broad Institute, Cambridge, MA 02142, USA; Division of Nephrology, Brigham and Women's Hospital, Boston, MA 02115, USA; Division of Nephrology, Department of Medicine, Columbia University Irving Medical Center, Columbia University, New York City, NY 10032, USA; Harvard Medical School, Boston, MA 02215, USA; Division of Nephrology, Department of Medicine, Beth Israel Deaconess Medical Center, Boston, MA 02215, USA; Harvard Medical School, Boston, MA 02215, USA; Division of Nephrology, Department of Medicine, Beth Israel Deaconess Medical Center, Boston, MA 02215, USA

**Keywords:** haplotype, *APOL1*, recombination, kidney disease

## Abstract

Black Americans are 3–4 times more likely to develop nondiabetic kidney disease than other populations. Exclusively found in people of recent African (AFR) ancestry, risk variants in Apolipoprotein L1 (*APOL1*) termed G1 and G2 contribute significantly to this increased susceptibility. Our group and others showed that a missense variant in *APOL1*, rs73885316 (p.N264K, “M1”), is remarkably protective against APOL1 kidney disease when co-inherited with the G2 risk allele. Since the distance between the M1 and G2 variants is only 367 base pairs, we initially suspected that 2 independent mutation events occurred to create non-risk M1-G0 and M1-G2 haplotypes. Here, we examined *APOL1* haplotypes in individuals of AFR ancestry from the 1000 Genomes Project, the Nephrotic Syndrome Study Network (NEPTUNE), and an ancient individual from the Allen Ancient Genome Diversity Project to determine how the M1-G2 haplotype arose. We demonstrate that M1 most likely first appeared on a non-risk G0 haplotype, and that a subsequent recombination event bypassed strong recombination hotspots flanking *APOL1* and occurred between p.N388Y389del on a G2 haplotype and M1 on a G0 haplotype to create the M1-G2 haplotype. Observing a recombination event within a small region between clinically relevant loci emphasizes the importance of studying the entire haplotype repertoire of a disease gene and the impact of haplotype backgrounds in disease susceptibility.

## Introduction

Black Americans are 3–4 times more likely to develop nondiabetic kidney disease than other populations (Laster *et al*. 2018), and exonic risk variants in Apolipoprotein L1 (*APOL1*) drive much of this increased susceptibility. APOL1 is a component of the trypanosome lytic factors responsible for the protection against African trypanosomiasis by *Trypanosoma brucei gambiense* and *Trypanosoma brucei rhodesiense* (Vanhamme *et al*. 2003; Pérez-Morga *et al*. 2005). Through co-evolution of host and parasite interactions, human *APOL1* acquired 2 coding variants in the past 10,000 years, termed “G1” [an allele containing 2 single nucleotide polymorphisms (SNPs) rs73885319 (Hg38 chr22:36265860 A > G, Ser342Gly) and rs60910145 (chr22:36265988 T > G, Ile384Met)] and “G2” [an allele tagged by a 6 base pair in-frame deletion rs71785313 (chr22:36265995 AATAATT > A, Asn388-Tyr389del)], that confer resistance to trypanosome infections and are only found in individuals of recent African (AFR) ancestry (Genovese, Friedman *et al*. 2010; Tzur *et al*. 2010; Thomson *et al*. 2014; Capewell *et al*. 2015). However, this evolutionary protection results in a kidney disease risk tradeoff, as biallelic carriers of *APOL1* risk variants (G1/G1, G1/G2, or G2/G2 high-risk genotypes) are at a 7–30-fold risk for hypertension-associated end-stage kidney disease (ESKD), focal segmental glomerulosclerosis, and HIV-associated nephropathy (Genovese, Friedman *et al*. 2010; Genovese, Tonna *et al*. 2010; Kopp *et al*. 2011). *APOL1* lacking the G1 or G2 defining variants is generally referred to as the non-risk “G0” genotype and detected in all populations (Friedman and Pollak 2021). Various mechanisms caused by the *APOL1* risk variants, such as perturbed lysosomal membrane integrity, cation efflux, and immune system activation, induce kidney cell death (Lan *et al*. 2014; Nichols *et al*. 2015; Shah *et al*. 2019; Bruno and Edwards 2021; Datta *et al*. 2024).

As approximately 15% of individuals with an *APOL1* high-risk genotype will develop ESKD (Friedman and Pollak 2021), identifying genetic modifiers for the risk variants can improve our understanding of the incomplete penetrance seen in APOL1 kidney disease. Recently, we and others discovered that a low frequency, missense modifier variant (“M1”) in *APOL1*, rs73885316 (chr22:36265628 C > A, p.N264K, gnomAD AFR allele frequency = 0.03), provides substantial protection against kidney failure when co-inherited with the G2 risk allele (Gupta *et al*. 2023; Hung *et al*. 2023; Gbadegesin *et al*. 2024). Cell toxicity conferred by the expression of both G1 and G2 variant *APOL1* constructs in vitro is significantly attenuated in the presence of the M1 variant, as the M1 variant is posited to reduce APOL1 pore function and presumably APOL1-associated toxicity (Lannon *et al*. 2019; Hung *et al*. 2023). As would be predicted, the M1 variant also decreases APOL1-mediated trypanolytic activity, possibly increasing susceptibility to trypanosome infections (Cuypers *et al*. 2016).

Different *APOL1* haplotype backgrounds, defined by combinations of variants inherited together, can elicit varying degrees of cytotoxicity, illustrating a need to understand haplotype formation for disease-modifying variants (Lannon *et al*. 2019; Gupta *et al*. 2024). As the distance between the M1 and G2 variants is 367 base pairs, we reckoned that 2 independent mutation events, instead of 1 or more recombination events, occurred to generate haplotypes containing the M1 variant (M1-G0 and M1-G2) given the proximity of the nucleotides affected (Cuypers *et al*. 2016; Gupta *et al*. 2023). Under this hypothesis, the existence of the M1 variant on both G0 and G2 haplotypes implies that either the M1 variant arose independently on G0 and G2 haplotypes or that G2 arose independently on G0 and M1-G0 haplotypes. To deduce how the M1-G2 haplotype formed, we examined *APOL1* haplotypes in individuals of AFR ancestry from the 1000 Genomes Project, the Nephrotic Syndrome Study Network (NEPTUNE) cohort, and an ancient individual from Africa (Gadegbeku *et al*. 2013; Lipson *et al*. 2020; Byrska-Bishop *et al*. 2022).

## Materials and methods

### 1000 Genomes Project haplotype analysis

Population demographics and phased variant call format (VCF) files aligned to the Hg38 reference genome from high coverage 30× whole genome sequencing (WGS) of 1000 Genomes Project individuals are accessible from the International Genome Sample Resource (IGSR) FTP site (https://ftp.1000genomes.ebi.ac.uk/vol1/ftp/) (Fairley *et al*. 2020; Byrska-Bishop *et al*. 2022). We selected all individuals of AFR ancestry and removed those with a known *APOL1* copy number variant from analyses to minimize haplotype artifacts (*n* = 881). We compiled 12 exonic variants, 44 intronic variants, five 3′UTR variants, and 8 intergenic variants that define our haplotypes (*n* = 69 variants, Supplementary Fig. 1 and Supplementary Table 1). The 12 exonic variants chosen overlap prior investigations on the in vitro cytotoxicity of specific *APOL1* coding haplotypes and risk variants, including the M1 variant, and variations within *APOL1* observed in modern and archaic humans (Thomson *et al*. 2014; Lannon *et al*. 2019; Gupta *et al*. 2024). The remaining 57 variants were identified based on a 1000 Genomes AFR unrelated allele frequency > 0.10 and localized to and positionally downstream of *APOL1* (full region examined Hg38 coordinates chr22:36253528-36270279), of which 29 variants were previously examined in Thomson *et al*. (2014). Monomorphic variants for AFR individuals were excluded in this analysis. We split the phased alleles and counted unique haplotypes observed in the cohort. Hierarchical clustering of haplotypes using hclust() of Jaccard distances for binary traits and visualizations of the various *APOL1* haplotypes were performed in R.

### NEPTUNE haplotype analysis

We inspected haplotypes from 171 unrelated NEPTUNE individuals with genotype-predicted AFR ancestry to detect haplotypes in a nondiabetic kidney disease cohort similarly found in the 1000 Genomes Project analysis. WGS data were aligned to the Hg38 reference genome and phased using Shapeit5 with the 1000 Genomes Project Hg38 30X WGS variant call set as the reference panel (Gadegbeku *et al*. 2013; Hofmeister *et al*. 2023). We queried the 69 variants leveraged in the 1000 Genomes Project haplotype analysis within the NEPTUNE cohort and subsequently visualized unique haplotypes in R.

### Ancient DNA (aDNA) haplotype query

Prepublication binary alignment maps (bams) from shotgun WGS (median coverage 4.9×) and supplementary sample metadata for 216 ancient individuals in the Allen Ancient Genome Diversity Project/John Templeton Ancient DNA Atlas are available from the David Reich Lab (Harvard Medical School, Department of Genetics, Boston, MA; https://reich.hms.harvard.edu/ancient-genome-diversity-project). Sequence data in this collection were aligned to the hs37d5 reference genome, an Hg19 reference with additional decoy regions. We examined the 69 variants of interest in 1 M1 variant carrier from this collection, who was excavated from Shum Laka, an archeological site in Cameroon, using the Interactive Genomics Viewer (IGV, Supplementary Fig. 6) (Thorvaldsdóttir *et al*. 2013; Lipson *et al*. 2020). Conversion of genomic coordinates from Hg38 to Hg19 for the variants was performed using the UCSC liftOver tool, confirming position annotations with the National Institutes of Health's dbSNP database (Sherry *et al*. 2001; Hinrichs *et al*. 2006). We manually curated the lifted positions in IGV, marking whether a position contained at least 1 alternate allele call at the respective position. We created a “combined” haplotype of the 69 variants for this carrier (M1 Hg19 coordinate chr22:36661674 C > A) determined as whether the individual carried a variant of interest since this data was not phased.

### Recombination frequencies

Recombination frequencies were obtained from the 1000 Genomes recombination rate track from the UCSC Genome Browser Repository (https://hgdownload.soe.ucsc.edu/gbdb/hg38/recombRate/) (1000 Genomes Project Consortium; Abecasis *et al*. 2010). Recombination rates surrounding *APOL1* and nearby genes were visualized using R.

### Linkage disequilibrium statistics

Pairwise LD statistics for the 69 variants examined in this study were visualized using the LDMatrix tool from the National Institute of Health's LDLink web server (https://ldlink.nih.gov/? tab=ldmatrix) from all AFR individuals with high coverage Hg38 WGS (Machiela and Chanock 2015).

## Results and discussion

**Fig. 1.**
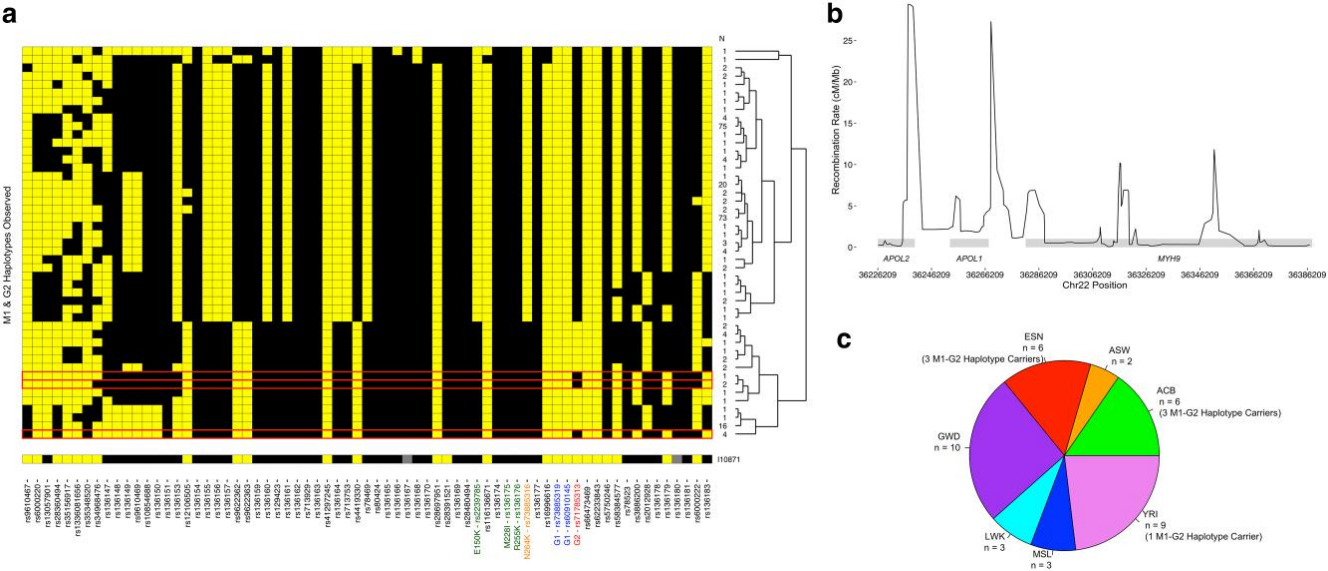
Haplotype analysis of 1000 Genomes Project AFR individuals. a) Haplotypes containing the *APOL1* M1 (p.N264K, rs73885316, label orange) and G2 (rs71785313, label red) variants observed in 881 AFR individuals from the 1000 Genomes Project high coverage 30× WGS collection. Positions with the alternate allele are black, while reference alleles are yellow. Three haplotypes (boxed in red) contain the M1 and G2 variants on the same haplotype. The G1 variant (rs73885319 and rs60910145) and variants that comprise EIK, KIK, and EMR haplotype backgrounds are labeled blue and green, respectively. The total counts of haplotypes detected and the hierarchical clustering of haplotypes are presented to the right of the table. The bottom haplotype is a “combined” haplotype from WGS of an ancient AFR individual I10871 from the Allen Ancient Genome Diversity Project that carried the M1 variant. Gray positions represent a variant with no sequencing reads available to make a genotype call. b) Sex-averaged recombination frequencies on chromosome 22 spanning *APOL2*, *APOL1*, and *MYH9* from 1000 Genomes Project individuals. c) Population distribution of individuals that carry M1-G0 and M1-G2 haplotypes (*n* = 39) from a). GWD, Gambian in Western Division; YRI, Yoruba in Ibadan, Nigeria; ASW, African Ancestry in Southwest US; ACB, African Caribbean in Barbados; ESN, Esan in Nigeria; MSL, Mende in Sierra Leone; LWK, Luhya in Webuye, Kenya.

*APOL1* haplotypes, defined by 69 phased variants (Supplementary Fig. 1 and Supplementary Table 1), from high coverage WGS of 881 individuals of AFR ancestry in the 1000 Genomes Project were used to investigate the co-inheritance of M1 and G2 (Byrska-Bishop *et al*. 2022). In this population, we identified 310 G0, 40 G1, and 36 G2 unique variant containing haplotypes (Supplementary Fig. 2). M1 appeared only on G0 and G2 haplotypes and not G1, as previously detected in cohorts of patients with or without kidney disease (Fig. 1a and Supplementary Fig. 3) (Gupta *et al*. 2023). Seven individuals carried the M1 and G2 variants on the same haplotype, encompassing 3 unique M1-G2 haplotypes. Moreover, our haplotype analysis highlighted M1-G0 and M1-G2 exclusive intronic SNPs, rs136155, rs136161, and rs713753, not seen in G1 or p.N264-G2 haplotypes. These SNPs label a G0 background not associated with other risk variant haplotypes.

Previous efforts studying *APOL1* haplotype backgrounds reported experimental differences in function and cytotoxicity contingent on the exonic variant structure at amino acid positions 150 (p.Glu150Lys, rs2239785), 228 (p.Met228Ile, rs136175), and 255 (p.Arg255Lys, rs136176) (Lannon *et al*. 2019; Winkler *et al*. 2022; Gupta *et al*. 2024). The “EIK” background facilitates podocyte death in vitro when tested with the risk variants, while the “KIK” and reference “EMR” backgrounds have minimal cytotoxic effects with these variants. To date, no G1 carriers have been discovered with a KIK background, while approximately 1% of G2 carriers have this background (Gupta *et al*. 2024). Here, we found p.N264-G2 haplotypes on an EIK background, while M1 variant exclusive haplotypes (including M1-G2 haplotypes) on a KIK background.

We performed hierarchical clustering of M1-G0, M1-G2, and p.N264-G2 haplotypes and leveraged recombination rate and linkage disequilibrium data to help identify the origin of the M1-G2 haplotype (Fig. 1a). Hierarchical clustering resulted with M1-G0 and M1-G2 haplotypes clustering while the remaining G2 haplotypes were separately grouped, indicating that M1-G2 haplotypes share a G0 background. Variants positionally upstream of the M1 variant in M1-G2 haplotypes are observed across M1-G0 haplotypes, whereas downstream of M1 is similar to p.N264-G2 haplotypes. Upon evaluating recombination frequencies from the 1000 Genomes Project around *APOL1*, we see increases at 6.19–27.28 cM/Mb at the 5′ and 3′ regions of *APOL1*, respectively (Fig. 1b), and variants localized to these regions are not in linkage disequilibrium (Supplementary Fig. 4) (1000 Genomes Project Consortium; Abecasis *et al*. 2010; Machiela and Chanock 2015). In particular, the M1 variant is in near linkage equilibrium with most of the variants examined, including the G2 variant, most likely due to the variant's low frequency in AFR populations. To confirm equivalent M1-G2 haplotype patterns, we inspected M1 and G2 containing haplotypes in 171 AFR patients with kidney disease from the NEPTUNE cohort (Gadegbeku *et al*. 2013). In this cohort, the frequencies of the G2 and M1 variants are 0.22 and 0.04, respectively, and we found 20 unique G2 and M1 haplotypes. Five individuals carried 1 M1-G2 haplotype equivalent to an M1-G2 haplotype in the 1000 Genomes Project cohort (Supplementary Fig. 5).

While we do not know when the M1-G2 haplotype first appeared, recent advances in sequencing ancient genomes can shed light on a variant's appearance in human evolution. We scanned the WGS of an ancient AFR individual dated 6058–5889 Bce from the archaeological site Shum Laka in northwest Cameroon (person I10871 from the Allen Ancient Genome Diversity Project). This ancient genome contained the M1 variant on a G0, KIK haplotype background like in the 1000 Genomes Project and NEPTUNE cohorts (Fig. 1a, bottom, and Supplementary Fig. 6) (Lipson *et al*. 2020). Furthermore, the existence of such an individual genome supports that the M1 variant has existed for at least 8,000 years in West Africa. In the 1000 Genomes Project cohort, individuals with the M1-G2 haplotype resided in Nigeria (ESN) and Barbados (ACB) (Fig. 1c). Notably, most M1 variant carriers inhabited West Africa, albeit the 1000 Genomes consortium predominately enrolled West African populations. Ko *et al*. (2013) saw the M1 variant in individuals from 6 African subgroups at a 6 and 5% allele frequency in Lemande and Mada (Cameroon), at an 8% frequency in both Borana and Sengwer (Kenya), and at a striking 34% allele frequency in Hadza and 5% Iraqw (Tanzania) (Ko *et al*. 2013). Only M1-G0 haplotypes were identified in their study. The frequency of the M1 variant may vary depending on the region, and more epidemiological screening is needed to discern its origins.

**Fig. 2.**
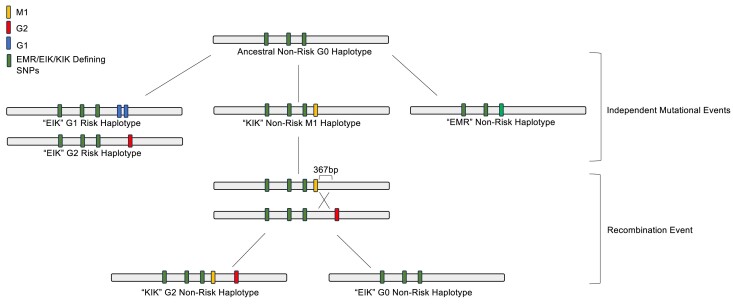
*APOL1* M1-G2 haplotype formation. Graphical representation of how *APOL1* M1-G2 haplotypes arose from an ancestral, non-kidney disease risk G0 haplotype. The haplotype backgrounds EMR, EIK, and KIK denote amino acid substitutions at positions 150, 228, and 255 (E150K, M228I, R255K). The EIK, KIK, and EMR haplotypes arose from an ancestral G0 haplotype, albeit the reference haplotype EMR is rarely seen in individuals of recent AFR ancestry. We suspect that the G1 (blue) and G2 (red) variants arose through independent mutations on an EIK background, while the M1 (orange) variant appeared on a KIK background. To have a haplotype containing the M1 and G2 variants on a KIK background, a recombination event within a 367 bp region must have occurred to generate the M1-G2 haplotype.

Based on the M1-G0 and M1-G2 exclusive intronic SNPs, the similar KIK background, the grouping of M1-G0 and M1-G2 haplotypes, and supporting recombination rate data around *APOL1*, we conclude that the M1 variant emerged first on the G0 haplotype, and a subsequent recombination event occurred with a G2 haplotype to create the M1-G2 haplotype (Fig. 2). Forming from a recombination event with a G0 haplotype instead of independent mutations on G0 and G2 haplotypes introduces additional complexities for understanding the M1 variant protective effect. If a G2 haplotype acquired the M1 variant independently, M1 could be the only difference on an ancestral G2 haplotype and mediate the protection against kidney disease. However, the M1-G2 haplotype carries a KIK G0 haplotype background and additional G0-specific variants that may influence *APOL1* expression or protein function. As increased *APOL1* expression is an additional mechanism for APOL1 kidney disease (Daneshpajouhnejad *et al*. 2022), several intronic SNPs within regulatory elements may influence *APOL1* expression levels. To evaluate the regulatory potential of a SNP, investigators harness resources like the Encyclopedia of DNA Elements (ENCODE) and RegulomeDB to understand if SNPs fall within candidate cis-regulatory elements (cCREs) and likely impact on gene regulation (Boyle *et al*. 2012; ENCODE Project Consortium; Moore *et al*. 2020). For example, intronic SNP rs136164 localizes to a distal enhancer-like regulatory element and is specific to M1-G0 and M1-G2 haplotypes but not G2 haplotypes (Supplementary Table 1).

Additional questions remain about the protective nature of M1 and the increased susceptibility to trypanosomiasis. Previous in vitro experiments highlighted abrogated lytic activity against *T. b. brucei* and *T. b. rhodesiense* for G1 APOL1 when the M1 was present, while M1 and G2 APOL1 maintained lytic activity (Cuypers *et al*. 2016). It is unclear how M1 and G2 APOL1 preserves lytic activity if the M1 variant is hypothesized to decrease APOL1 pore function. Moreover, no M1-G1 haplotype in humans has been detected. The likely explanation for this observation is no recombination events have occurred to phase the M1 and G1 variants, as the M1 and G1 variants are 232 base pairs apart. Alternatively, since the G1 variant is more concentrated in West Africa than G2, and the G1 variant effectively lyses *T. b. rhodesiense* and *Trypanosoma brucei* with a serum resistance antigen (Genovese, Friedman *et al*. 2010; Thomson *et al*. 2014), negative selection pressures could eliminate potential M1-G1 haplotypes arising. However, insufficient evolutionary time may have passed to see the M1 variant at more common frequencies to determine immediate selection pressures.

Observing a recombination event in such a small region between clinically impactful loci is surprising and provides a unique opportunity to investigate the consequence of modifying variants in disease susceptibility loci. More clinical and epidemiological screening of this variant should be conducted to evaluate its role in our evolutionary history and epidemiology of kidney disease. Additional functional testing of the M1 variant against trypanosomes and the mechanism of cytotoxicity in mammalian cells is needed to understand the APOL1 channel ablation. This recombination event underscores the importance of studying the entire spectrum of *APOL1* haplotypes and not the risk variants individually.

## Supplementary Material

jkae290_Supplementary_Data

## Data Availability

Data from the 1000 Genomes Project are publicly available from the International Genome Sample Resource's FTP site. Whole genome sequencing data from the NEPTUNE cohort are available through dbGaP accession number phs003210.v1.p1. The authors affirm that all data necessary for confirming the conclusions of this article are represented fully within the article and its tables and figures. Supplemental material available at G3 online.
