# Supplementary material for: The APOL1 p.N264K variant is co-inherited with the G2 kidney disease risk variant through a proximity recombination event
Source: G3 (Bethesda). 2024 Dec 10;15(2):jkae290. doi: 10.1093/g3journal/jkae290 (PMC11797048; doi:10.1093/g3journal/jkae290)
Supplement: jkae290_Supplementary_Data [file jkae290_supplementary_data.zip › Supplemental_Figures_G3-2024-405425.docx]

The *APOL1* p.N264K variant is co-inherited with the G2 kidney disease risk variant through a proximity recombination event

**Supplementary Material**

Christopher A. Simeone,^1,2^ Michelle T. McNulty,^3^ Yask Gupta,^4^ Giulio Genovese,^1,5^ Matthew G. Sampson,^1,3^ Simone Sanna-Cherchi,^4^ David J. Friedman,^1,2^ Martin R. Pollak ^1,2^

**Affiliations:**

^1^ Harvard Medical School, Boston, MA, 02215.

^2^ Division of Nephrology, Department of Medicine, Beth Israel Deaconess Medical Center, 99 Brookline Ave, Boston, MA, 02215.

^3^ Division of Pediatric Nephrology, Boston Children’s Hospital, 320 Longwood Ave, Boston, MA, 02215

^4^ Division of Nephrology, Department of Medicine, Columbia University Irving Medical Center, Columbia University, 622 West 168th Street, New York City, NY 10032

^5^ Stanley Center, Broad Institute of MIT and Harvard, 77 Avenue Louis Pasteur, Boston, MA, 02215

**Keywords:** Haplotype, *APOL1*, Recombination, Kidney Disease

**Corresponding Authors:** Christopher Simeone, PhD csimeone@bidmc.harvard.edu

Martin Pollak, MD [mpollak@bidmc.harvard.edu](mailto:mpollak@bidmc.harvard.edu)

David Friedman, MD [dfriedma@bidmc.harvard.edu](mailto:dfriedma@bidmc.harvard.edu)

**Table of Contents**

**Figure S1.** SNP positions examined in this study 3

**Figure S2.** All *APOL1* haplotypes observed in 1000 Genomes Project AFR individuals 4

**Figure S3.** *APOL1* G1 containing haplotypes observed in 1000 Genomes Project AFR individuals 5

**Figure S4.** Linkage disequilibrium statistics for 69 variants examined in this study 6

**Figure S5.** *APOL1* M1 and G2 containing haplotypes observed in NEPTUNE AFR patients 7

**Figure S6.** The M1 variant detected in ancient DNA 8

**Figure S1**


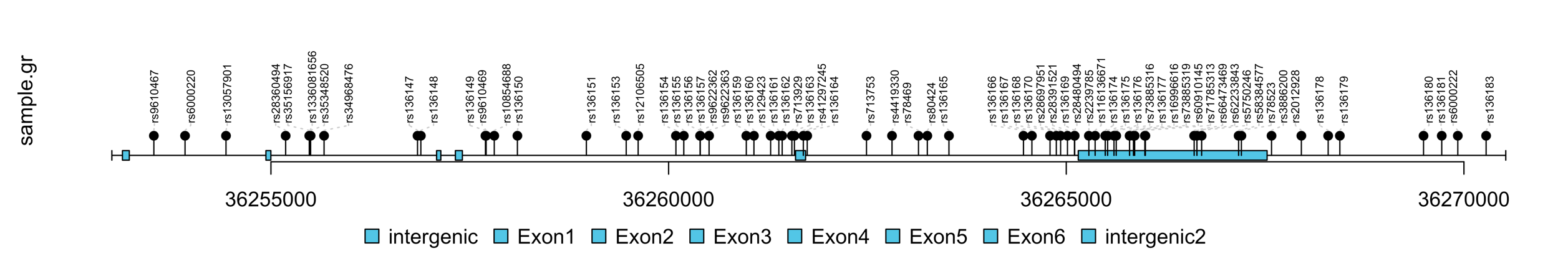


**Figure S1. SNP positions examined in this study.** We studied 69 phased variants localized to and downstream of *APOL1* on Chromosome 22. Blue rectangles indicate exonic boundaries within *APOL1*.

**Figure S2**

**
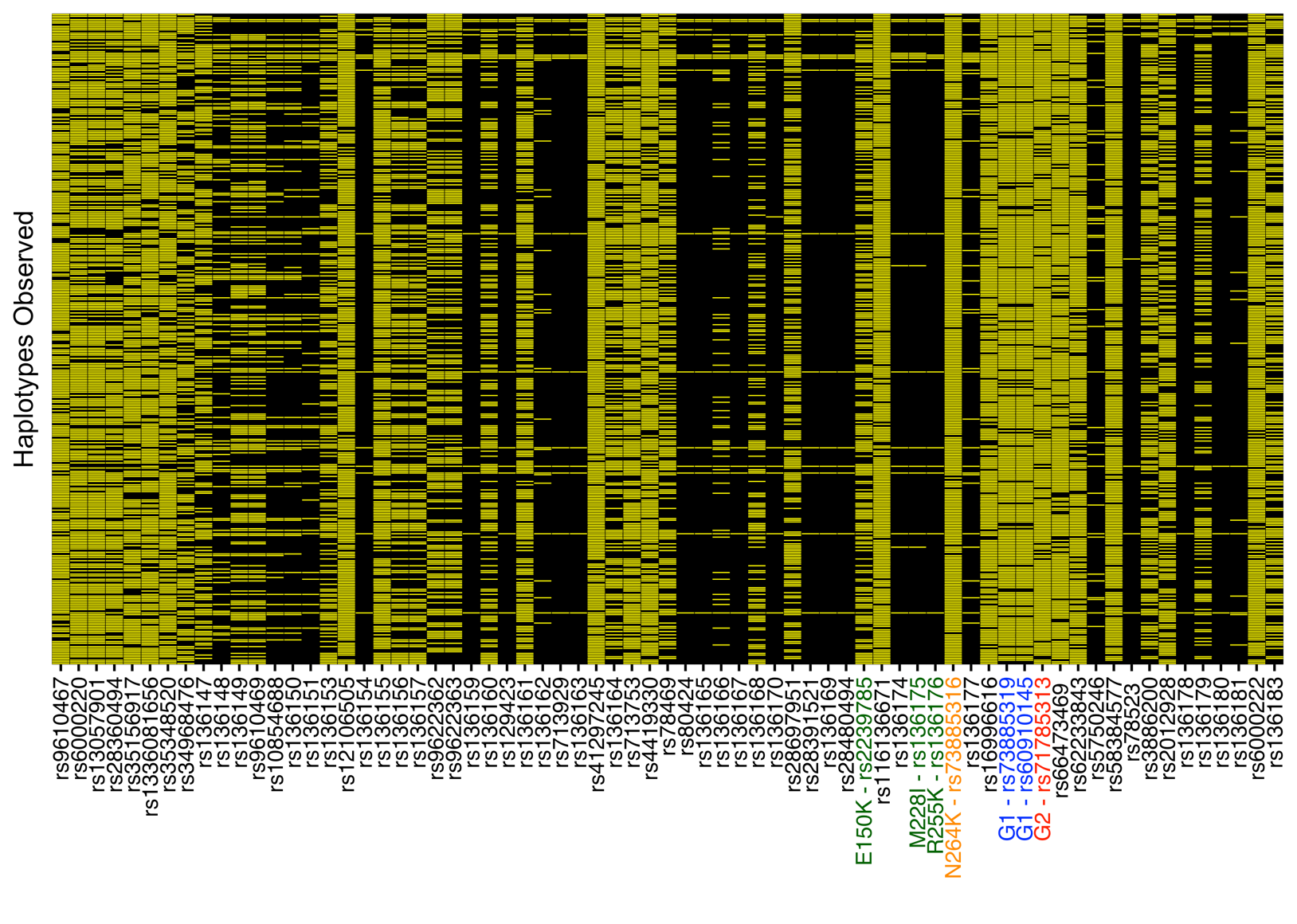
**

**Figure S2. All *APOL1* haplotypes observed in 1000 Genomes Project AFR individuals.** Three hundred and eighty-six unique haplotypes were isolated from AFR individuals in the 1000 Genomes Project high coverage 30X whole genome sequencing collection (n=881). Positions with the alternate allele are black, while reference alleles are yellow. The M1 (p.N264K, rs73885316), G1(rs73885319 and rs60910145), and G2 (rs71785313) variants are x-axis labeled orange, blue, and red, respectively. Positions that make up known *APOL1* haplotype backgrounds are x-axis labeled green.

**Figure S3**

***
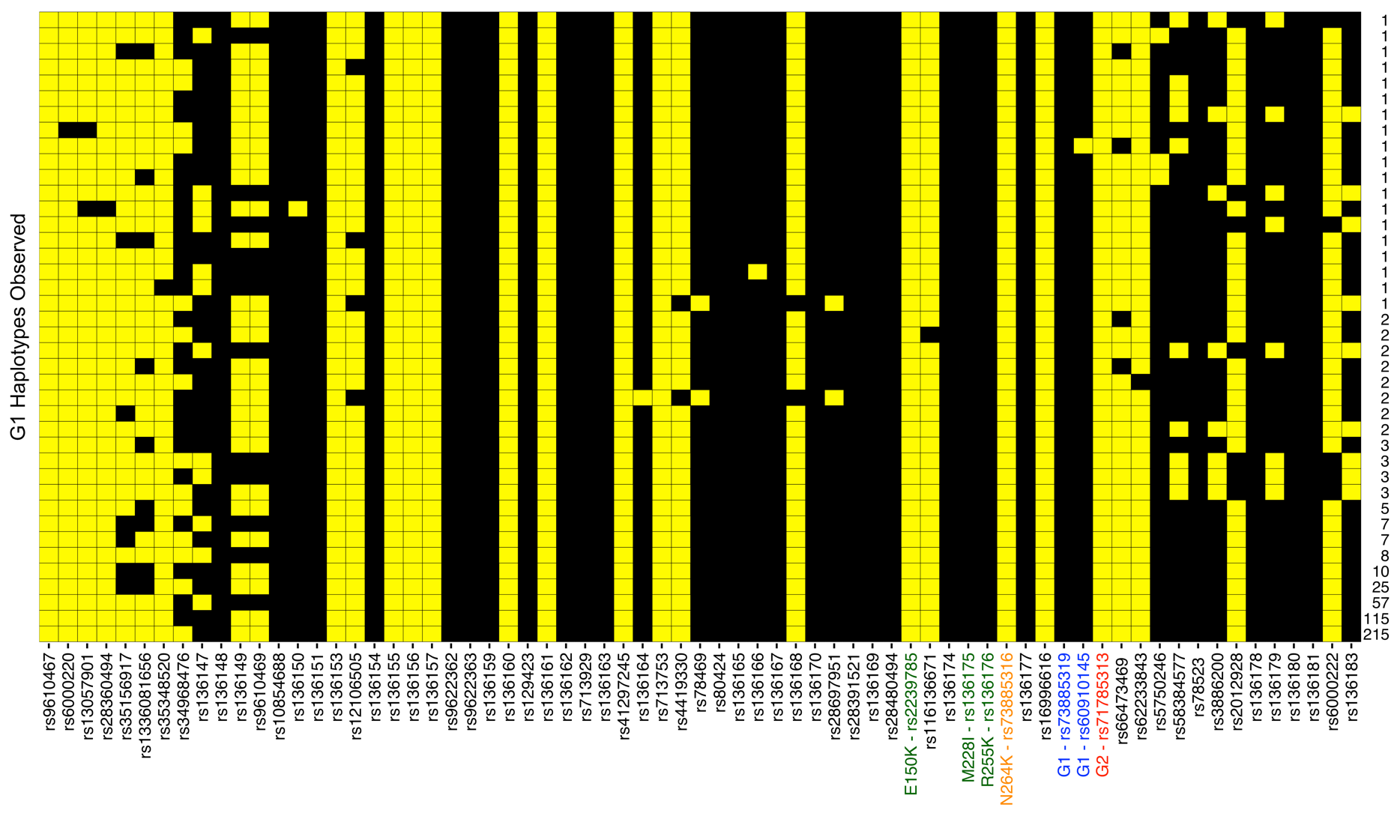
***

**Figure S3. *APOL1* G1 containing haplotypes observed in 1000 Genomes Project AFR individuals.** Forty unique G1-containing haplotypes were isolated from AFR individuals in the 1000 Genomes high coverage 30X whole genome sequencing collection (n=881). Positions with the alternate allele are black, while reference alleles are yellow. Haplotype counts within this population are presented on the right of each haplotype. The M1 (p.N264K, rs73885316), G1(rs73885319 and rs60910145), and G2 (rs71785313) variants are x-axis labeled orange, blue, and red, respectively. Positions that make up known *APOL1* haplotype backgrounds are x-axis labeled green.

**Figure S4**

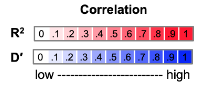


**Figure S4. Linkage disequilibrium statistics for 69 variants examined in this study**. Heatmap matrix of pairwise LD statistics for the 69 variants in this study from all AFR individuals in high coverage whole genome sequencing from the 1000 Genomes Project using the LDMatrix tool from the National Institute of Health’s LDLink web server.

**Figure S5**

**Figure S5. *APOL1* M1 and G2 containing haplotypes observed in NEPTUNE AFR patients.** Twenty unique M1 and G2 haplotypes were isolated from AFR patients in the Nephrotic Syndrome Study Network (NEPTUNE) whole genome sequencing collection (n=171). Positions with the alternate allele are black, while reference alleles are yellow. We identified one unique M1-G2 haplotype (*boxed in red*) that was similarly seen in the 1000 Genomes Project. Haplotype counts within the population are presented on the right of each haplotype. The M1 (p.N264K, rs73885316), G1(rs73885319 and rs60910145), and G2 (rs71785313) variants are x-axis labeled orange, blue, and red, respectively. Positions that make up known *APOL1* background haplotypes are x-axis labeled green.

**Figure S6.**

**Figure S6. The M1 variant detected in ancient DNA.** IGV view of the M1 variant (p.N264K, rs73885316, Hg19 chr22:36661674) in shotgun whole genome sequencing from the ancient individual I10871 in the Allen Ancient Genome Diversity Project / John Templeton Ancient DNA Atlas. Sequencing data in this collection was aligned to the hs37d5/hg19 reference genome.
